# Two new species of endemic Ecuadorean Amaryllidaceae (Asparagales, Amaryllidaceae, Amarylloideae, Eucharideae)

**DOI:** 10.3897/phytokeys.48.4399

**Published:** 2015-04-02

**Authors:** Alan W. Meerow, Lou Jost, Nora Oleas

**Affiliations:** 1 USDA-ARS-SHRS, National Germplasm Repository, 13601 Old Cutler Road, Miami, Florida 33158, USA; 2 EcoMinga Foundation, Via a Runtun, Baños, Tungurahua, Ecuador; 3 Universidad Tecnológica Indoamérica, Centro de Investigación de la Biodiversidad y Cambio Climático, Machala y Sabanilla, Quito, Ecuador

**Keywords:** Bulb, Amaryllidaceae, Andes, South America, *Stenomesson*, *Eucharis*

## Abstract

New species of the genera *Stenomesson* and *Eucharis* (Amaryllidaceae) are described from Ecuador. *Stenomesson ecuadorense* is the second species of the genus reported from that country, and the only endemic one. It is related to *Stenomesson miniatum* and *Stenomesson campanulatum*, both from Peru, with which it shares orange flower color and the fusion of the staminal corona to the perianth tube. It differs from *Stenomesson miniatum* by the non-urceolate perianth, from *Stenomesson campanulatum* by its shorter stamens and longer perianth, and from both by its lower montane, cloud forest habitat. *Eucharis ruthiana*, found in the vicinity of Zamora, is related to *Eucharis moorei* from which it differs by the narrower leaves and tepals; short, deeply cleft staminal corona; the long teeth on either side of the free filaments; the narrowly subulate, incurved free filaments; and the shorter style. The green mature fruit and campanulate floral morphology place it in Eucharis subg. Heterocharis.

## Abstract (es)

Nuevas especies de los géneros *Stenomesson* y *Eucharis* (Amaryllidaceae) se describen para Ecuador. *Stenomesson ecuadorense* es la segunda especie del género reportada de ese país y la única endémica. Está relacionada con *Stenomesson miniatum* y *Stenomesson campanulatum*, ambos de Perú, con la que comparte el color naranja de la flor y la fusión de la copa estaminal al tubo del perianto. Se diferencia de *Stenomesson miniatum* por el perianto no urceolado, de *Stenomesson campanulatum* por sus estambres más cortos y perianto más largo, yde ambos por su hábitat en bosque nuboso montano de baja altitud. *Eucharis ruthiana*, que se encuentra en las cercanías de Zamora, está relacionada con *Eucharis moorei*, de cual difiere por las hojas y tépalos estrechos, copa estaminal corta y profundamente hendida, los dientes largos a ambos lados de los filamentos libres, filamentos libres encurvos y ligeramente subulados, y el estilo más corto. El fruto maduro verde y morfología floral campanulada lo posicionan en *Eucharis* subgénero *Heterocharis*.

## Introduction

Ecuador is a major center of diversity for the Andean tetraploid clade of American Amaryllidaceae, specifically genera in the tribe Eucharideae (Meerow 1990), a monophyletic group characterized by pseudopetiolate leaves and the loss of the gene *ndhF* from the plastid genome (Meerow 2010; Meerow et al. 2000). In this paper, we describe two new species in the tribe, *Stenomesson ecuadorense*, and *Eucharis ruthiana*, both endemic to Ecuador.

## Materials and methods

No specimens matching these new species were observed in herbarium collections in Ecuador (with the exception of the single specimen cited below under *Stenomesson ecuadorense*) housed at QCA, QCNE, HUTI, nor encountered by the first author in collections examined over the past 30 years at GB, K, MO, and NY.

## Taxonomy

### Stenomesson ecuadorense

Taxon classificationPlantaeAsparagalesAmaryllidaceae

Meerow, Oleas & Jost sp. nov.

urn:lsid:ipni.org:names:77146127-1

Fig. 1

#### Diagnosis.

**Figure 1.**
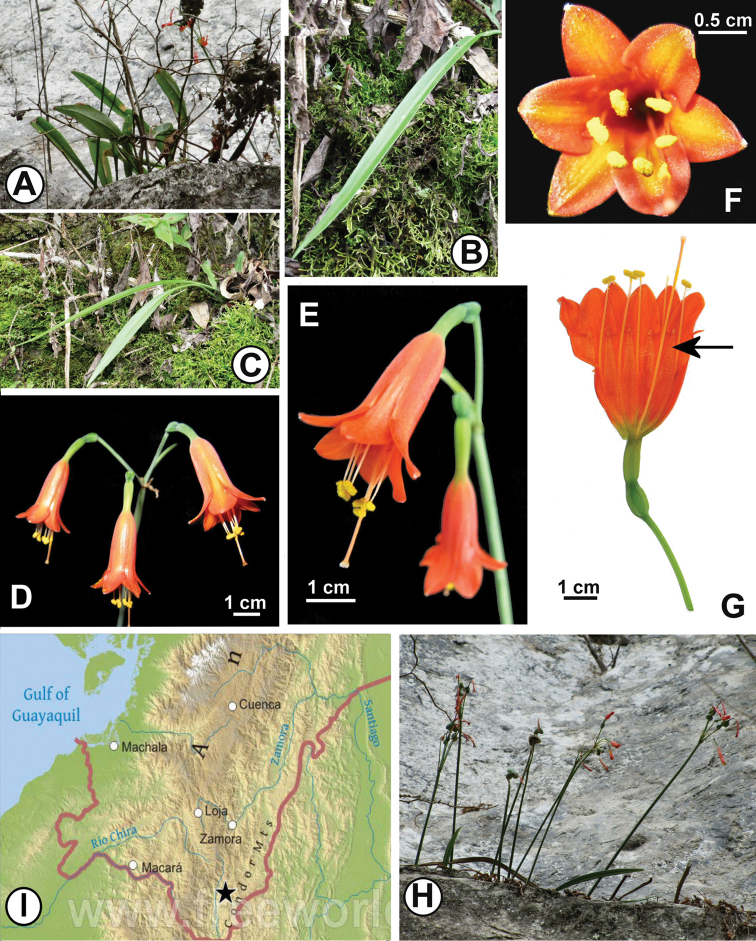
*Stenomesson ecuadorense*. **A–C** Plants in habit on limestone cliff **D–G** Inflorescence and flowers. Arrow in **G** denotes androecial teeth interposed between the free filaments **H** Plants in fruit in habitat **I** Distribution in Ecuador (black star). Map courtesy of www.freeworldmaps.net. The apparent yellowish stripes in Fig. 1F are artifacts of camera flash reflectance and are not visible by eye.

**Figure 2.**
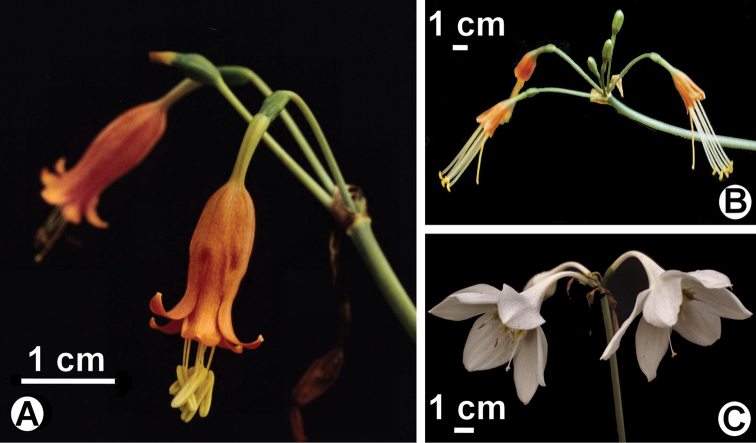
Species closely related to the new taxa described in this paper. **A** *Stenomesson miniatum* (*Meerow* 1148, FTG) **B** *Stenomesson campanulatum* (*Meerow* 2445 (NA) **C** *Eucharis moorei* (*Meerow & Meerow* 1141, FLAS).

*Stenomesson ecuadorense* (Fig. 1) appears closely related to the Peruvian *Stenomesson miniatum* (Herb.) Ravenna (1978; Fig. 2A) and *Stenomesson campanulatum* Meerow (1985; Fig. 2B) by flower color and adherence of the staminal corona to the floral tube, consisting of six long teeth interposed between the free filaments, but differs from them by the moist habitat, occurence on limestone and relatively low elevation (Table 1). *Stenomesson miniatum* has an urceolate corolla. *Stenomesson campanulatum* has a non-patent limb and long-exserted stamens. The perianth of *Stenomesson ecuadorense* has flaring tepals as does *Stenomesson miniatum*, but is long campanulate in morphlogy.

**Table 1.** Contrasting features of *Stenomesson ecuadorense*, *Stenomesson campanulatum* and *Stenomesson miniatum*.

Character	*Stenomesson ecuadorense*	*Stenomesson campanulatum*	*Stenomesson miniatum*
Pedicel length	19–28 mm	25–40 mm	12–25 mm
Perianth morphology	Long-campanulate	Campanulate	Urceolate
Limb morphology	Flaring	Non-patent	Flaring, apically recurved
Exsertion of stamens beyond perianth	ca. 10 mm	25–30 mm	5–10 mm
Elevation	< 1300 m	2200–2600 m	2500–3500 m

#### Type.

ECUADOR: Zamora-Chinchipe, Tapala, on limestone cliffs above Río San Luis, near its confluence with the Río Numbala; 4°32.478'S, 79°03.985'W, ca. 1295 m elevation, 18 March 2006 (observed in flower and fruit; specimens made from flowering cultivated plants 10 Apr 2012), *Lou Jost* 7949 (Holotype: QCA!, Isotypes: QCNE!, HUTI!, MO!, NA!, NY!).

#### Description.

Geophytic, hysteranthous, perennial from tunicate bulbs. Bulbs globose to ovoid, offsetting readily, tunics brown, 2–4 cm diam, apically forming a neck 1–5 cm long. Leaves (Fig. 1A–C) 1–2 per bulb, glabrous, 18.5–30 cm long, tapering at base to a ca. 5 cm long hemiterete pseudopetiole; lamina lanceolate, sometimes slightly falcate, 14–14.5 × 1.8–3.0 cm wide at the middle, midrib inconspicuous adaxially, prominent abaxially, acute at apex, Royal Horticultural Society Color Chart (RHSCC, Royal Horticultural Society 1995) green 137A adaxially, 137D abaxially. Inflorescence scapose, 1–4 flowered, scape 25–30 cm tall, 3.7–3.9 mm diam, terete, glaucous, solid for most of its length with a narrow lumen apically, terminated by 2 marcescent ovate-lanceolate bracts enclosing the buds in the early stages of elongation, 20.9–21.6 mm long, 3.4–3.6 mm wide at base, 6 mm wide at middle, acute at apex. Flowers (Fig. 1D–G) pendulous via the spreading pedicels and curvature of the tube, 3.6–4 cm long from base of ovary to limb apex; pedicels 19–28 × ca. 0.5 mm. Perianth (Fig. 1D, E) actinomorphic, cylindrical proximally, distally campanulate, consisting of six tepals in two whorls, fused below the throat into a tube that is 2.7–3 mm diam, cylindrical, and green in the proximal 1–1.2 cm, constricting to 1.8–2.3 mm in its distal 3–4 mm before abruptly dilating to 7.3 mm and becoming orange (RHSCC orange red 33A). Limb of free tepals (Fig. 1F) spreading ca. 60° from the throat, 1.7–1.9 cm wide; outer tepals 9.8–10.6 mm × 4.8–5.6 mm (at middle), acute, with a white, papillose apiculum; inner tepals 7.5–8.5 mm long, 6.5–7 mm wide, minutely apiculate. Stamens joined at base into an inconspicuous membranous staminal corona in the form of six 0.8–1.0 cm long lanceolate, acute teeth, fused to the perianth tube except for the apical 1.0 mm of each tooth (Fig. 1G), with the filaments inserted between; free filaments filiform, light orange for their proximal third, then white in their distal 2/3, 1.7–1.8 cm long, exserted ca. 1 cm beyond the limb; anthers 1.8–2 mm long, oblong, dorsifixed, introrse; pollen yellow. Style 3.5–3.7 cm long, exserted 5–6 mm past stamens, orange, fading to light orange distally; stigma obscurely tri-lobed, 1–1.4 mm wide. Ovary ellipsoid, ca. 6.7 mm long, ca. 3.2 mm wide, ovules 20 or more per locule, axile in placentation. Mature fruit (Fig. 1H) a trigonous, papery, tri-loculicidal capsule ca. 1 cm long and 1.5 cm wide; seeds numerous, papyraceous, flattened, shortly obliquely winged, with a dark brown phytomelanous testa.

#### Distribution and ecology.

*Stenomesson ecuadorense* is so far only known from the type locality in southern Ecuador (Fig. 1I) where it grows on what appear to be limestone cliffs above the Río San Luis, just below 1300 m. It was first found by LJ in 2006 and subsequently examined in the field by AM and NO in 2009. As so far known, the species is restricted to these cliffs where it grows in cracks, crevices and narrow shelves on the rock where pockets of humus accumulate. We estimate the population that we observed to consist of several hundred individuals. The full extent of occurrence is not yet known.

#### Etymology.

The species is named for the nation of Ecuador, to where it so far appears to be endemic.

#### Additional material examined.

ECUADOR: Zamora Chinchipe, same locality as type, 1254 m elev., 04°33'38"S, 79°04'39"W, flowering. 23 June 2014, Pérez A. J., et al. 7260 (QCA).

#### Notes.

The genus *Stenomesson* Herb. (*sensu* Meerow et al. 2000) includes about 15 spp., and is primarily found in Peru, with only *Stenomesson aurantiacum* Herb. previously reported from Ecuador (Meerow 1990). The genus usually occurs in seasonally dry, grassy vegetation or at the margins of cloud forest above 2000 m elevation, but is also found in Peruvian inter-Andean valleys below 2000 m (Meerow and van der Werff 2004), and the loma formations along the coast of Peru. The new species, *Stenomesson ecuadorense*, is found below 1300 m elevation in relatively wet habitat.

*Stenomesson ecuadorense* appears closely related to *Stenomesson miniatum* (Peru, Bolivia; Fig. 2A) and *Stenomesson campanulatum* Meerow (Peru; Fig. 2B) by the orange flower color and fusion of the staminal corona to the floral tube, but differs from them (Table 1) by the unusual limestone habitat and relatively low elevation. *Stenomesson miniatum* has an urceolate corolla, and is always found above 2000 m in elevation to as high as 3500 (unpubl. herbarium data). *Stenomesson campanulatum* has a non-patent limb and long-exserted stamens (Meerow 1985). The perianth of *Stenomesson ecuadorense* has flaring tepals as does *Stenomesson miniatum*, but is long campanulate in morphology. It is only the second species of the genus (*sensu* Meerow et al. 2000) reported from Ecuador, and so far the only endemic one.

### Eucharis ruthiana

Taxon classificationPlantaeAsparagalesAmaryllidaceae

Jost, Oleas & Meerow sp. nov.

urn:lsid:ipni.org:names:77146128-1

Fig. 3

#### Diagnosis.

*Eucharis ruthiana* appears closely related to *Eucharis moorei* (Baker) Meerow (Meerow 1987a), placing it in Eucharis subg. Heterocharis Meerow, characterized by large campanulate, fragrant flowers, numerous ovules per locule, and green mature capsules (Meerow 1989). It differs from this species by the narrower leaves and tepals, mild (vs. strong) fragrance, deeply cleft staminal corona with long marginal teeth, the incurved free filaments, and the short style (Table 2). From the putative hybrid Eucharis × grandiflora Planch. & Lind., (1853) it differs by the long staminal teeth and its fertility.

**Table 2.** Contrasting features of *Eucharis ruthiana* and *Eucharis moorei*.

Character	*Eucharis ruthiana*	*Eucharis moorei*
Leaf/width ratio	3:1	< 2:1
No. flowers	8–16	4–7
Tepal width	15–19 mm	17–27 mm
Staminal corona	Deeply incised	Connate for most of its length
Length of staminal teeth	7.5–8 mm	2.5–3 mm
Habit of free staminal filament	Incurved	Straight
Style length	2.3–2.6 cm	6–7 cm
Floral Fragrance	Mild	Strong

#### Type.

ECUADOR: Zamora-Chinchipe, near Zamora, on rocky soil in the understory of lower montane forest ca. 1100 m elevation, June 2006, *Jost 8278* (Holotype: QCA!, Isotypes: QCNE!).

#### Description.

**Figure 3.**
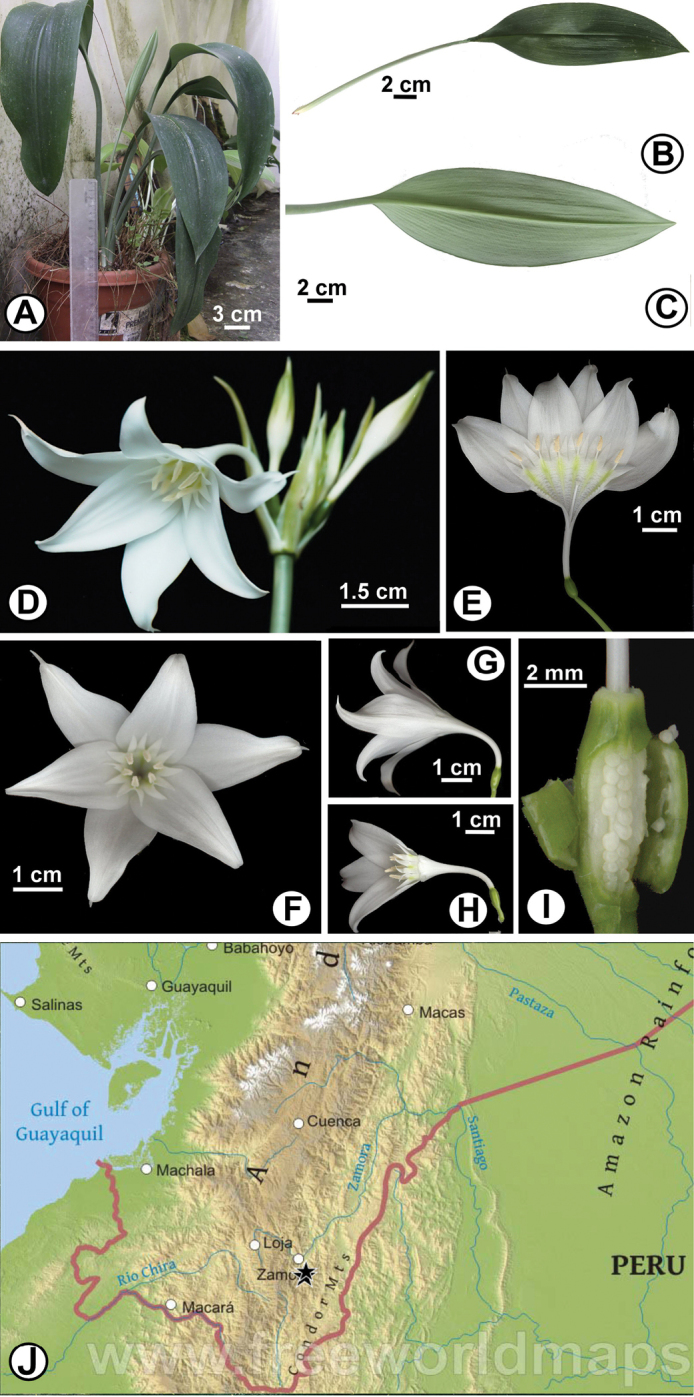
*Eucharis ruthiana*. **A** Plant in cultivation **B–C** Leaves **B** Adaxial view **C** Abaxial view **D–H** Flowers **D** Upper portion of inflorescence showing flower habit **E** Flower cut and spread to show staminal corona **F** Dorsal-ventral view of limb showing the spread of the androecium **G** Lateral view **H** Lateral view with three tepals removed to show androecium **I** Ovary dissected to show numerous, superposed, globose ovules **J** Distribution of *Eucharis ruthiana* in Ecuador (black stars). Map courtesy of www.freeworldmaps.net.

Geophytic, evergreen perennial from tunicate bulbs, tunics reddish brown, thin; immature bulb ca. 3 cm × 2.5 cm. Leaves (Fig. 3A–C) 2–5 per bulb, glabrous, tapering at base to a 19–18 cm long pseudopetiole that is 6–8 mm thick; lamina elliptical, ca. 28 cm × 9 cm, dark green adaxially and shallowly plicate, light green abaxially, acute at apex. Inflorescence scapose, scape 8–16 flowered, ca. 40 cm tall, 4 mm diam, terete, glaucous, solid, terminated by 2 greenish-white, eventually marcescent ovate-lanceolate bracts enclosing the buds in the early stages of elongation, ca. 3 cm long, ca. 5 mm wide at base, acute at apex. Flowers (Fig. 3D–G) slightly declinate, white, mildly fragrant, 4.5–5.0 cm long; pedicels 2–6 cm long, the last flowers to reach anthesis with the longest, with a narrow bracteole subtending each. Perianth (Fig. 3D, E) actinomorphic, funnelform-campanulate, consisting of six tepals in two whorls, fused below the throat into a slightly curved tube that is 15–2.0–2.2 cm long 2.7–3 mm diam, white for its entire length, cylindrical in the proximal 1.3–1.5 cm, then funnel-form distally, dilating to 0.85–10.0 mm at throat, limb spreading ca. 60° from the throat, 5–6 cm wide; outer tepals 28–36 mm × 15 mm, acute, with a white, ca. 3 mm long papillose apiculum; inner tepals 27–35 × 17–19 mm, minutely apiculate. Stamens joined at base into a 2.5–3.0 × 1.5–2.0 cm staminal corona deeply divided into six pairs of lanceolate, free, tooth-like processes, such that only the lower 2.6–3.0 mm of the corona is connate, stained yellowish-green along the filamental traces, most prominently on the inside surface; each tooth 7.5–8 mm long, acute at the apex and slightly recurved above the middle, with the six free filaments inserted between the teeth of each pair; free filaments narrowly subulate, slightly incurved towards center of the corona, 3–4 mm long, anthers oblong, 3–4 × < 1 mm, white, dorsifixed, introrse; pollen white. Style 2.3–2.6 cm long, not exserted past stamens, white; stigma tri-lobed, papillate, ca. 2 mm wide. Ovary ellipsoid, 4–5 mm long, ca. 3.2 mm wide, ovules 16–20 per locule, superposed, axile in placentation. Ripe fruit green, seed globose, bluish-black.

#### Distribution and ecology.

*Eucharis ruthiana* is only known from the type locality and a private reserve in southern Ecuador (Fig. 3I), in lower montane rain forest where it grows on stony soil in the understory of dense forest at ca. 1100 m elevation. A large population occurs on the Copalinga private reserve near Zamora according to the property owner.

#### Etymology.

The species is named in honor of the late Ruth Moore, ardent supporter of conservation efforts in Ecuador.

#### Notes.

Eucharis subg. Heterocharis was erected by Meerow (1989), even though it appeared paraphyletic in his cladistic analysis of morphological characters. The large, fragrant flowers; numerous ovules per locule, and mature green fruits were considered symplesiomorphic for the genus. The subgenus previously included only two fertile species, *Eucharis sanderi* Baker (1883), endemic to the Chocó region of Colombia, and *Eucharis moorei* (Baker) Meerow (1987a), found on both the eastern and western declivities of the Ecuadorean Andes. The group also contains two apparently sterile taxa, Eucharis × grandiflora Planch. & Lind. (1853), a putative hybrid of *Eucharis moorei* and *Eucharis sanderi*, found in southern Colombia and northern Ecuador (Meerow 1989), most often in cultivation, and *Eucharis amazonica* Lind. ex Planch. (Meerow and Dehgan 1984). The latter, most commonly found in the lower Huallaga Valley of Peru, never sets seed, has a triploid-derived chromosome number (2*n* = 68), and impaired pollen fertility (Nagalla 1979; Meerow 1987b). *Eucharis ruthiana* appears most closely related to *Eucharis moorei*, but is easily separable (Table 2) by the narrower leaves and tepals, the deeply cleft staminal cup with long marginal teeth, the short, nearly filiform, incurved free filaments, and the relatively short style.

## Supplementary Material

XML Treatment for Stenomesson ecuadorense

XML Treatment for Eucharis ruthiana
